# Nickel-Titanium Alloys in Endodontics: A Narrative Review

**DOI:** 10.7759/cureus.105033

**Published:** 2026-03-11

**Authors:** Pierluigi Valente, Lapo Sbrenna, Andrea Sbrenna, Francesco Valente

**Affiliations:** 1 School of Dentistry, Vita-Salute San Raffaele University, Milan, ITA; 2 School of Dentistry, Alma Mater Studiorum - University of Bologna, Bologna, ITA; 3 Private Practice, Humanis Dental Center, Perugia, ITA; 4 Oral Implantology, San Damiano Dental Clinic, Rome, ITA

**Keywords:** austenitic phase, endodontic instrumentation, endodontic shaping, glide-path, heat-treated niti, martensitic phase, nickel-titanium (niti) instruments, root canal biomechanics, r-phase

## Abstract

The introduction of nickel-titanium (NiTi) rotary instruments has profoundly transformed modern endodontics, enabling safer and more conservative shaping of complex root canal systems. Despite technological advances, unexpected instrument fracture remains a relevant clinical concern, highlighting the importance of understanding the metallurgical and biomechanical mechanisms governing instrument behavior. This review aims to critically analyze the metallurgical evolution, biomechanical performance, and clinical implications of NiTi instruments, with particular emphasis on heat-treated alloys, phase transformations, shaping kinematics, and stress management during canal preparation. A non-systematic narrative review methodology was adopted. Relevant scientific literature addressing NiTi metallurgy, cyclic fatigue resistance, torsional stress, corrosion phenomena, glide path preparation, and rotary versus reciprocating kinematics was analyzed, integrating experimental, metallurgical, and clinical studies selected for their conceptual relevance to contemporary endodontic practice. The clinical performance of NiTi instruments results from the interaction between alloy metallurgy, geometric design, and operative protocols. Thermomechanical treatments increase martensitic phase presence at clinical temperatures, enhancing flexibility and cyclic fatigue resistance. However, surface defects, corrosive environments, and stress accumulation remain critical factors in instrument failure mechanisms, indicating that material properties alone do not determine clinical safety. Contemporary root canal shaping should be interpreted as a biomechanical stress management process rather than simple canal enlargement. Integration of metallurgical knowledge with operative strategies represents a fundamental prerequisite for safer and biologically conservative endodontic treatment.

## Introduction and background

The primary objective of endodontic therapy is to perform tridimensional cleaning, shaping, and disinfection of the root canal system while preserving the original anatomical pathway and the tooth's structural integrity. Achieving this balance has historically been a major challenge due to the anatomical variability and complexity of root canal curvature [1].

The introduction of nickel-titanium (NiTi) instruments marked a paradigm shift in endodontics, allowing improved canal centering and reduced procedural errors compared with stainless steel instruments. The unique mechanical behavior of NiTi derives from reversible solid-state phase transformations between austenite and martensite, enabling superelasticity and shape memory properties. However, the widespread adoption of NiTi instrumentation introduced new biomechanical challenges, particularly unexpected instrument fracture [2]. This phenomenon stimulated extensive research into metallurgical structure, fatigue behavior, and stress distribution during canal preparation [3]. Over the last two decades, innovation has progressively moved from geometric design optimization toward metallurgical engineering. Advanced thermomechanical treatments have modified phase transformation temperatures, promoting martensitic and R-phase stability at body temperature and improving flexibility and cyclic fatigue resistance. Simultaneously, the development of reciprocating kinematics and the increasing emphasis on glide path preparation have demonstrated that clinical safety depends not solely on material properties but on the interaction between alloy behavior and operative strategy. Therefore, root canal shaping is increasingly interpreted as a dynamic biomechanical process involving continuous interaction between anatomy, instrument metallurgy, and mechanical loading conditions [4].

Endodontic NiTi instruments are manufactured from a near-equiatomic alloy composed of approximately 55% nickel and 45% titanium by weight. The distinctive behavior of this alloy arises from reversible phase transformations occurring in the solid state, which differentiate NiTi from conventional dental alloys [5]. Three principal crystallographic phases are involved: austenite (B2 cubic structure), martensite (B19′ monoclinic structure), and R-phase (intermediate rhombohedral phase). The relative distribution of these phases depends on temperature and applied mechanical stress, resulting in a dynamically adaptive material during clinical use [6].

The austenitic phase is stable above the austenite finish (Af) temperature and is characterized by higher stiffness and elastic modulus. NiTi instruments operating predominantly in this phase exhibit superelastic behavior through stress-induced martensitic transformation when negotiating canal curvature, allowing recovery of the original shape but potentially leading to greater cyclic stress accumulation during continuous rotation [7]. Martensite exhibits lower elastic modulus and greater deformability than austenite. Heat-treated NiTi instruments that stabilize martensite at clinical temperatures therefore demonstrate increased flexibility, reduced restoring forces, and improved resistance to cyclic fatigue, particularly in severely curved canals [8]. The R-phase represents an intermediate crystallographic state in NiTi alloys in which deformation occurs through crystallographic twinning, allowing stress accommodation without permanent lattice distortion. Instruments containing an R-phase component therefore exhibit reduced restoring forces and improved fatigue resistance and canal tracking [9].

NiTi mechanical properties depend on transformation temperatures: martensite start (Ms), martensite finish (Mf), austenite start (As), and Af. When intracanal temperature approximates Af, minor thermal or mechanical variations may alter phase distribution during instrumentation. Consequently, NiTi instruments behave as adaptive materials whose mechanical response changes dynamically under clinical conditions [10]. The evolution of NiTi instruments primarily reflects advances in thermomechanical processing rather than major compositional changes. Early-generation instruments were predominantly austenitic, whereas contemporary alloys undergo controlled heating and cooling cycles to modulate phase transformation behavior. Memory Wire (M-Wire) technology introduced a multiphase microstructure containing martensite, austenite, and R-phase, improving cyclic fatigue resistance compared with conventional NiTi [11]. Subsequent developments, including controlled memory (CM) alloys and proprietary heat treatments, further enhanced flexibility and fatigue resistance. This transition represents a conceptual shift from geometry-driven design toward metallurgically engineered instruments optimized for biomechanical stress distribution [12].

Biomechanical implications of NiTi metallurgy

Instrument behavior during shaping results from the interaction between alloy phase composition and mechanical loading [13]. Austenitic instruments generally exhibit higher cutting efficiency but may accumulate greater cyclic stress during continuous rotation. Instruments with higher martensitic content demonstrate greater flexibility and improved adaptation to canal curvature. The presence of R-phase contributes to more gradual stress dissipation during deformation [14]. Clinical safety, therefore, depends on the relationship between metallurgy and operative strategy rather than on instrument design alone [15].

The aim of this narrative review is to provide an integrated interpretation of the metallurgical principles of NiTi alloys and their role in the evolution of endodontic instruments, by correlating phase transformation behavior with biomechanical performance and clinical implications in contemporary endodontics. The literature discussed in this review was identified through PubMed and Scopus searches without time restrictions, using keywords related to NiTi metallurgy, phase transformation behavior, heat‑treated alloys, cyclic fatigue, torsional resistance, corrosion, and biomechanical aspects of endodontic instrumentation. Studies were selected based on conceptual relevance to the metallurgical and biomechanical themes addressed. As a narrative review, this work does not follow Preferred Reporting Items for Systematic Reviews and Meta-Analyses (PRISMA) methodology or include a formal risk‑of‑bias assessment, and these methodological limitations are acknowledged; however, the narrative approach was intentionally adopted to allow a broader conceptual synthesis of the interaction between NiTi metallurgical properties and the biomechanical behavior of endodontic instruments.

## Review

The metallurgical principles described above provide the foundation for understanding how NiTi instruments behave under clinical mechanical loading. The following section analyzes how these phase transformations influence biomechanical performance during root canal shaping. The studies discussed in this review were not selected to provide an exhaustive or systematic synthesis of the literature, but rather to illustrate the key scientific concepts that have progressively shaped the current understanding of NiTi endodontic instrumentation. Articles were therefore included based on their conceptual relevance to metallurgical behavior, biomechanical performance, and clinical stress management during canal preparation. Experimental, metallurgical, and clinical investigations were integrated to construct a coherent interpretative framework that links material science with operative endodontic strategies. This narrative approach aims to emphasize the evolution of underlying principles rather than to compare isolated outcomes among different instrument systems.

The clinical performance of NiTi endodontic instruments is fundamentally governed by the metallurgical behavior of the alloy rather than by geometric design alone. The macroscopic shaping ability observed during root canal instrumentation represents the clinical expression of microstructural phase transformations occurring within the material under thermal and mechanical stress conditions. NiTi alloys used in endodontics are near-equiatomic alloys composed of approximately 55-56% nickel and 44-45% titanium by weight. Their unique mechanical behavior derives from reversible solid-state phase transformations responsible for shape memory and superelasticity, properties extensively described in shape memory alloy theory. From a crystallographic perspective, NiTi may exist in three principal structural configurations: austenite (B2 cubic phase), martensite (B19′ monoclinic phase), and R-phase (intermediate rhombohedral phase) [16].

The relative distribution of these phases depends on temperature and applied stress, making NiTi a dynamically adaptive material during clinical use. Understanding these phase-dependent behaviors is essential because the clinical performance of NiTi instruments cannot be interpreted solely through mechanical outcomes. Rather, shaping efficiency, safety, and fracture resistance emerge from the continuous interaction between metallurgical phase transformations and biomechanical loading conditions encountered during canal preparation. Consequently, the following sections interpret instrument behavior as the clinical manifestation of material science principles operating under dynamic intracanal stresses.

Austenitic phase, superelasticity, and restoring force

At temperatures above the Af temperature, NiTi instruments predominantly exhibit an austenitic structure characterized by higher stiffness and elastic restoring force. Conventional NiTi instruments function mainly in this phase at body temperature. During canal instrumentation, curvature-induced stress triggers a stress-induced martensitic transformation, allowing significant elastic deformation followed by recovery upon stress release. This phenomenon, known as superelasticity, enables improved canal centering and reduced transportation compared with stainless steel instruments. However, increased restoring forces associated with austenitic dominance may lead to higher stress concentration during continuous rotation, contributing to cyclic fatigue accumulation [17].

Martensitic phase, flexibility, and stress dissipation

The martensitic phase presents a lower elastic modulus and enhanced deformability. Instruments containing a higher martensitic fraction demonstrate increased flexibility and reduced restoring forces, allowing passive adaptation to canal curvature. Modern heat-treated instruments are engineered to stabilize martensite at clinical temperatures by modifying transformation temperatures through thermomechanical processing. Experimental investigations have demonstrated significantly improved cyclic fatigue resistance in martensitic-dominant alloys, particularly in severely curved canals [18]. Importantly, martensitic behavior does not eliminate fracture risk, but delays crack initiation and propagation by distributing mechanical stress more evenly [19].

R-phase and transitional mechanics

Between austenite and martensite, NiTi may transition through an intermediate R-phase characterized by reduced elastic modulus and lower transformation energy requirements. The transformation sequence austenite → R-phase → martensite enables gradual accommodation of deformation through crystallographic twinning mechanisms, limiting permanent lattice dislocations. The presence of the R-phase has been associated with improved flexibility and smoother stress redistribution during cyclic loading. Clinically, instruments exhibiting R-phase behavior demonstrate reduced shape-restoring forces and improved anatomical tracking ability (Table 1).

**Table 1:** Metallurgical phases of NiTi alloys and their biomechanical implications in endodontic instrumentation. NiTi: nickel-titanium; Af: austenite finish

Phase	Crystallographic structure	Mechanical characteristics	Metallurgical behavior	Biomechanical effect during shaping	Clinical implications
Austenite	B2 cubic phase	Higher stiffness and restoring force	Stable above Af with stress-induced martensitic transformation	Higher stress concentration during rotation	Efficient cutting but higher cyclic fatigue risk
Martensite	B19′ monoclinic phase	High flexibility and deformability	Stabilized by thermomechanical treatments	Stress dissipation and reduced restoring force	Improved adaptation and fatigue resistance
R-phase	Rhombohedral intermediate phase	Reduced stiffness and transformation energy	Transitional phase via crystallographic twinning	Smoother stress redistribution	Enhanced anatomical tracking

Transformation temperatures and clinical behavior

The mechanical properties of NiTi instruments are strongly influenced by the transformation temperatures Ms, Mf, As, and Af. When body temperature approaches Af, small thermal or mechanical variations may alter phase distribution during instrumentation. Consequently, the same instrument may behave differently depending on intracanal temperature, irrigation conditions, rotational speed, and mechanical loading [20]. Experimental investigations have confirmed that increasing temperature significantly reduces cyclic fatigue resistance of NiTi instruments, likely due to stabilization of the austenitic phase and increased material stiffness [21]. This concept supports the modern interpretation of NiTi instruments as adaptive materials whose mechanical behavior evolves dynamically during clinical use.

Evolution of heat-treated NiTi alloys

Technological evolution in NiTi instruments has primarily involved metallurgical engineering rather than changes in chemical composition. Thermomechanical treatments have progressively modified phase stability to optimize biomechanical performance. Conventional NiTi instruments were predominantly austenitic at clinical temperatures, combining cutting efficiency with relatively high stiffness. The introduction of heat-treated alloys, including M-Wire and controlled memory wire (CM-wire) technologies, increased the proportion of martensite and R-phase present during clinical operation. M-Wire alloys exhibit a multiphase microstructure that significantly enhances cyclic fatigue resistance compared with conventional NiTi. Subsequent CM-wire technologies further reduced elastic memory, enabling instruments to be pre-curved and to conform passively to canal anatomy. Modern heat-treated systems, therefore, represent a conceptual shift from geometry-driven design toward metallurgically engineered stress control [22].

Metallurgy as a determinant of biomechanical stress distribution

The evolution of NiTi metallurgy has transformed endodontic instrumentation into a process governed by stress management rather than purely mechanical enlargement. Austenitic instruments tend to generate higher restoring forces and localized stress concentration, whereas martensitic and heat-treated instruments distribute mechanical loads more uniformly along the instrument length. This redistribution delays fatigue accumulation and improves safety margins during shaping procedures. Accordingly, shaping performance should be interpreted as the interaction between canal anatomy and material phase behavior rather than as a function of instrument design alone [23].

Cyclic fatigue and mechanisms of instrument fracture

Instrument separation remains one of the principal complications of rotary instrumentation. Cyclic fatigue occurs when an instrument rotates within a curved canal, undergoing repeated tensile and compressive stress cycles that progressively generate microcracks until sudden fracture occurs. Heat-treated alloys significantly increase fatigue resistance; however, fractographic analyses demonstrate that microscopic manufacturing defects may serve as stress concentrators capable of initiating premature failure even in advanced alloys [24]. Surface treatments such as electropolishing reduce micro-irregularities and improve crack resistance, highlighting the combined importance of bulk metallurgy and surface integrity. Thus, improved metallurgy enhances resistance but does not abolish intrinsic material vulnerability.

Chemical environment and corrosion effects

The endodontic environment further influences instrument longevity. Sodium hypochlorite, the most widely used irrigant, may induce corrosive surface alterations, including pitting corrosion and oxide layer modification. Recent electrochemical studies suggest that corrosion acts as a fatigue accelerator rather than an isolated degradation process, weakening surface integrity and promoting crack propagation under cyclic loading [25]. Clinical durability, therefore, depends not only on alloy composition but also on irrigation protocols and instrument handling [26-28].

Glide path preparation as a biomechanical safety strategy

The creation of a glide path represents a critical factor in reducing mechanical stress during instrumentation. Establishing a reproducible pathway allows rotary instruments to operate under reduced torsional load and frictional resistance [29]. NiTi glide path instruments have demonstrated improved canal centering and reduced anatomical alterations compared with manual preflaring techniques [30]. Micro-computed tomography analyses further confirm superior preservation of original canal morphology when a mechanical glide path is established [31]. From a biomechanical perspective, glide path preparation converts shaping from forced penetration into guided instrumentation, significantly enhancing procedural safety.

Rotational versus reciprocating kinematics

Instrument kinematics plays a central role in stress modulation. Reciprocating motion reduces continuous tensile stress accumulation, thereby increasing cyclic fatigue resistance compared with continuous rotation. Comparative studies show that reciprocating and rotary systems have similar shaping abilities and both preserve canal anatomy when appropriate protocols are followed. However, rotary motion has been associated with a higher incidence of postoperative pain [32]. Reciprocating endodontic files generally demonstrate greater resistance to cyclic fatigue compared with instruments operating in continuous rotary motion. Moreover, when evaluated in canals with double curvature, reciprocating systems have been shown to maintain superior fatigue resistance even under more complex anatomical conditions, indicating an enhanced ability to withstand repeated mechanical stresses during root canal preparation [33]. Rather than representing competing technologies, rotary and reciprocating movements should be interpreted as different strategies for mechanical energy distribution within the instrument [34,35].

Shaping ability and anatomical preservation

The biological objective of root canal shaping is the preservation of the original canal trajectory. Modern NiTi systems maintain canal centering even in complex anatomies due to the combined influence of alloy flexibility and instrument design. Anatomical preservation emerges from the balance among metallurgical flexibility, kinematic strategy, glide path preparation, and operator control.

Toward a biomechanical paradigm of endodontic shaping

Current evidence suggests that endodontic instrumentation should be interpreted within a biomechanical framework integrating materials science and clinical technique. Metallurgy determines material behavior, kinematics modulates stress application, glide path preparation distributes mechanical load, and operator strategy integrates these variables into a controlled shaping process. Modern endodontics may therefore be interpreted as a form of applied material biomechanics in which successful shaping results from controlled stress management rather than aggressive mechanical enlargement.

## Conclusions

The evolution of NiTi instruments has progressively transformed root canal shaping into a biomechanically guided procedure. Clinical performance results from the interaction between metallurgy, instrument design, and operative protocols. Heat-treated alloys improve flexibility and fatigue resistance; however, fracture prevention primarily depends on biomechanical stress management through appropriate clinical strategies. Collectively, current evidence supports interpreting contemporary endodontic shaping as an application of material biomechanics, in which NiTi instruments function as adaptive systems responding dynamically to clinical stress conditions and enabling safer, biologically conservative canal preparation.
